# Community health workers at the dawn of a new era

**DOI:** 10.1186/s12961-021-00761-7

**Published:** 2021-10-12

**Authors:** Joseph M. Zulu, Henry B. Perry

**Affiliations:** 1 Health Promotion and Education Department, School of Public Health, University of Zambia, Lusaka, Zambia; 2 Department of International Health, Johns Hopkins Bloomberg School of Public Health, Baltimore, MD USA

## Abstract

**Background:**

There is now rapidly growing global awareness of the potential of large-scale community health worker (CHW) programmes not only for improving population health but, even more importantly, for accelerating the achievement of universal health coverage and eliminating readily preventable child and maternal deaths. However, these programmes face many challenges that must be overcome in order for them to reach their full potential.

**Findings:**

This editorial introduces a series of 11 articles that provide an overview highlighting a broad range of issues facing large-scale CHW programmes. The series addresses many of them: planning, coordination and partnerships; governance, financing, roles and tasks, training, supervision, incentives and remuneration; relationships with the health system and communities; and programme performance and its assessment. Above all, CHW programmes need stronger political and financial support, and this can occur only if the potential of these programmes is more broadly recognized. The authors of the papers in this series believe that these challenges can and will be overcome—but not overnight. For this reason, the series bears the title “Community Health Workers at the Dawn of a New Era”. The scientific evidence regarding the ability of CHWs to improve population health is incontrovertible, and the favourable experience with these programmes at scale when they are properly designed, implemented, and supported is compelling. CHW programmes were once seen as a second-class solution to a temporary problem, meaning that once the burden of disease from maternal and child conditions and from communicable diseases in low-income countries had been appropriately reduced, there would be no further need for CHWs. That perspective no longer holds. CHW programmes are now seen as an essential component of a high-performing healthcare system even in developed countries. Their use is growing rapidly in the United States, for instance. And CHWs are also now recognized as having a critically important role in the control of noncommunicable diseases as well as in the response to pandemics of today and tomorrow in all low-, middle-, and high-income countries throughout the world.

**Conclusion:**

The promise of CHW programmes is too great not to provide them with the support they need to achieve their full potential. This series helps to point the way for how this support can be provided.

There is growing interest globally in large-scale community health worker (CHW) programmes. This is based on the increasing evidence of their potential to (1) contribute towards attaining local, national, and global health goals, including universal health coverage, and to (2) tap into the one of the critical resources for improving health in low-income settings—the communities themselves [1–3]. CHWs extend promotive, preventive, and curative health services into communities through community-level approaches while also fostering collective community action and local accountability [4–7]. Countries that have prioritized and invested in well-planned and properly supported large-scale CHWs—particularly Bangladesh, Brazil, Iran, Ethiopia, and Nepal—have been leaders in improving the health of their populations [8–16]. The gains in these countries and in many other countries include improvements in ending readily preventable child and maternal deaths, along with important contributions to the control of HIV, tuberculosis, malaria, and other infectious diseases [17]. The potential of CHWs to mitigate the burden of noncommunicable diseases and strengthen primary healthcare more broadly is now becoming even more widely appreciated [3, 18].

Despite growing momentum to build stronger large-scale CHW programmes, these programmes face many challenges at present [8, 19–21]. The challenges include lack of adequate funding, fragmented programming because of multiple external donors—each with a specific disease focus, inadequate supervision, lack of continuous performance assessments and improvements [22–24]. Further, integration of many large-scale CHW programmes with the community and with health systems has not been optimal [23, 25]. These challenges affect the quality and sustainability of services delivered by CHWs [19, 23].

While there have been increased calls to develop and implement large-scale CHW programmes, there has been little comprehensive documentation of these challenges, including guidance on how large-scale CHW programmes can be strengthened to better contribute to meeting health goals [23]. A notable exception was the comprehensive monograph produced in 2014 by Perry, Crigler, and Hodgins and colleagues entitled *Developing and Strengthening Community Health Worker Programs at Scale: A Reference Guide and Case Studies for Program Managers and Policy Makers* [26].

The supplement which follows here builds on this book. It is led by the same core group that led the production of the 2014 monograph: Henry Perry, Steve Hodgins, Lauren Crigler, Simon Lewin, Claire Glenton, Karen LeBan, Christopher Colvin, and Muhammad Mahmood Afzal.

The supplement contains 11 papers, all of which focus on major issues that need to be considered for CHWs to reach their full potential. The papers are based on lessons from case studies of large well-established CHW programmes, published literature, and the authors’ extensive experience. Key thematic areas covered in the papers include (1) tensions confronting CHW programmes [27]; (2) planning, coordination, and partnerships [28]; (3) programme governance [29]; (4) programme financing [30]; (5) roles and tasks [31]; (6) recruitment, training, and continuing education [32]; (7) supervision [33]; (8) incentives and remuneration [34]; (9) CHWs’ relationships with the health system and with the community [35]; (10) programme performance and its assessment [36]; and a concluding article on (11) CHWs leading the way to “Health for All” [37].

Large-scale CHW programmes face many challenges which affect their performance [19, 23, 38]. In the first paper of the series, “Introduction and Tensions Confronting Large-scale CHW Programmes” [27], Hodgins et al. identify concerns relating to the role of CHWs. They note that CHWs and their programmes face conflicting needs which are not easily resolved. In their paper on “Roles and Tasks” [31], Glenton et al. suggest that planners should address challenges with CHW roles by assessing whether the recommended roles and tasks are considered acceptable and appropriate by their target population, the CHWs themselves, and those who support them. The process of designing roles and tasks should be guided by research evidence and global experience as well as by the experiences, needs, and concerns of local communities and health workers. Other factors that need to be considered include the qualifications required and the training and continuing education needed in order to ensure quality performance and safe practices.

To better respond to their evolving and, in many instances, their gradually expanding roles, there is also a need to develop and implement systematic strategies for recruiting and training CHWs, including continuing education. In their paper, “Recruitment, Training, and Continuing Education” [32], Schleiff et al. recommend that effective training approaches cover relevant content, make use of appropriate technical training tools, update CHWs with additional knowledge as needed, and engage CHWs with other health workers during their training. They emphasize that improved and innovative training approaches can enhance the professionalism, quality, and performance of CHWs and contribute to effective scale-up.

In their paper on “Planning, Coordination, and Partnerships” [28], Afzal et al. note that many CHW programmes are not well integrated and synchronized with local health systems and local health needs. This is partly because such programmes are often centred on individual projects and implemented as vertical programmes with separate funding mechanisms. This situation has often led to gaps and fragmentation in service delivery. Poor planning, coordination, and failed partnerships can be better addressed by establishing and strengthening national coordination mechanisms for all categories of human resources for health, and by bringing in CHWs, CHW programmes, and their advocates as new, full-fledged partners in the planning and coordination processes for a national health workforce strategy.

The location of CHW programmes at the interface between the health system and communities presents a series of special challenges. In their paper on “Programme Governance” [29], Lewin et al. note that governance may be a challenge, since CHW programmes involve a wide range of stakeholders with diverse interests and power. Further, CHW programmes often lack comprehensive policy guidance, regulations and laws, governance structures, and clarity with regard to who will implement decisions. Strengthening governance systems requires collectively and systematically clarifying existing governance arrangements (which are often ambiguous) and specifying how they can be adapted and improved to better meet local needs.

Many large-scale CHW programmes also lack effective supervision. Furthermore, there is no universal “best approach” for CHW supervision. Effective and supportive supervision may help CHWs perform better by improving motivation, clarifying roles and tasks, and ensuring availability of needed tools and supplies, appropriate knowledge and skills, and a safe work environment [39]. In their paper on “Recent Advances in Supervision” [33], Carpenter et al. identify five categories of supervision, namely external supervision, community supervision, group supervision, peer supervision, and dedicated supervision. Improving supervision may be achieved by scaling up local innovations in supervision that suit the local context as well as by adapting novel approaches that have proven effective elsewhere.

Another element that is critical to strengthening the performance of large-scale CHW programmes is adequate financing. In their paper on “Programme Financing” [30], Masis et al. argue that though vital, financing has not received sufficient emphasis in the academic literature on CHW programmes. The authors stress the need for domestic government funding for CHWs. One way of helping to expand funding for CHW programmes is by increasing domestic political will for CHW programmes. Monitoring CHW programme expenditures in comparison with expenditures for facility-based primary healthcare services and for hospital services can help make the case for adequate funding commitments. Because of their cost-effectiveness in improving population health, CHW programme expenditures should be prioritized for expenditures as health systems evolve and mature.

Insufficient funding has led to inadequate and inconsistent incentives for CHWs, a situation that has contributed to low work motivation. Colvin et al., in their paper entitled “Incentives and Remuneration” [34], describe a broad range of financial and nonfinancial incentives that can be effective in motivating CHWs. The paper affirms the call in the recent guidelines for CHWs released by WHO in 2018 [40] for CHWs to receive a financial package that corresponds to their job demands and their complexity, the number of hours worked, and the length of training.

Given that CHW programmes are located at the interface between the health system and the community, it is important that they be well integrated into each in order for them to be maximally effective. In their paper on “CHWs’ Relationships with the Health System and Communities” [35], LeBan et al. note that building such relationships is not easy due to widely differing expectations, needs, and capacities. To facilitate functional interactions and relationships, there is a need for committed and competent leadership at all levels of the health system, clarity regarding the roles of all actors, and enhanced skills in communication and engagement with the community.

To further improve the performance of CHW programmes, there is a need to assess the performance of both individual CHWs and of the overall programme, including its contribution to community-level outcomes. Such evidence on performance, in turn, needs to be used to guide innovations and improvements. In their paper on “CHW Programme Performance and Its Assessment” [36], Kok et al. note that given the complexity of CHW programmes, it is important that assessment processes be based on data drawn from a variety of sources. Assessments should examine both the effectiveness of CHW programmes and the underlying, contextual factors that affect programme performance. CHW programmes need to be in a dynamic state of continual improvement.

CHW programmes are playing an increasingly important role in achieving national and global health goals, including the Sustainable Development Goals of universal health coverage. In the final and concluding paper of the series, “CHWs Leading the Way to ‘Health for All’” [37], Perry et al. note that to strengthen CHW programmes and help them better contribute to meeting health goals, it is important to pay attention to the most pressing challenges facing CHW programmes, the most important of which include inadequate financing, lack of supplies, low compensation of CHWs, and inadequate supervision. The authors argue that strong CHW programmes can serve as the foundation of effective health systems and should not continue to be an underfunded afterthought.

In summary, this series of papers provides a critical overview of large-scale CHW programmes, their challenges, and their potential. We believe that the papers make a compelling case that a serious commitment to such programmes will reap enormous benefits for population health—especially for the neediest and most vulnerable.

We join with the authors of these papers in calling for the “evidence–practice” gap to be closed, by investing in community programmes to increase the coverage of low-cost basic and essential preventive and curative interventions that communities and CHWs in low-income countries can be well equipped to provide. This evidence–practice gap is no longer morally acceptable. By building stronger CHW programmes and working constructively with communities on actions they can take, this gap can be narrowed, saving millions of lives.

## Data Availability

Not applicable.

## Abbreviation

CHW: Community health worker
